# Formulation, Optimization and In Vivo Evaluation of Fucoidan-Based Cream with Anti-Inflammatory Properties

**DOI:** 10.3390/md19110643

**Published:** 2021-11-17

**Authors:** Ekaterina D. Obluchinskaya, Olga N. Pozharitskaya, Elena V. Flisyuk, Alexander N. Shikov

**Affiliations:** 1 Murmansk Marine Biological Institute of the Russian Academy of Sciences (MMBI RAS), Vladimirskaya, 17, Murmansk 183010, Russia; obluchinskaya@gmail.com (E.D.O.); olgapozhar@mail.ru (O.N.P.); 2 Department of Technology of Pharmaceutical Formulations, St. Petersburg State Chemical Pharmaceutical University, Prof. Popov, 14a, Saint-Petersburg 197376, Russia; elena.flisyuk@pharminnotech.com

**Keywords:** anti-inflammatory, cream, drug release, formulation development, fucoidan, topical application

## Abstract

Fucoidan is a polysaccharide found in brown alga with glorious potential for pharmacological activities, among which its anti-inflammatory properties have gained meaningful attention. Due to several advantages of formulations for topical application, this study aimed to develop and optimize a fucoidan-based cream formulation and to investigate its anti-inflammatory potential after topical application in vivo. Fucoidan from *Fucus vesiculosus* L. was used. The cream base consisting of olive oil and Kolliphor RH40 was optimized followed by in vitro agar diffusion and drug release studies. The fucoidan-based cream with 13% Kolliphor P 407, 1% Transcutol P, and 5% PEG400 showed good spreadability, washability, and colloidal stability, and it did not irritate the skin. The kinetics of fucoidan release from the optimized cream exhibited the best fit to the Korsmeyer–Peppas and Higuchi models with *R*^2^ > 0.99. Fucoidan release was controlled by drug diffusion and anomalous transport provided by the optimized cream base. The formulation was stable and provided high fucoidan release after storage for 1 year. Topical application of the fucoidan-based cream dose-dependently inhibited carrageenan-induced edema and ameliorated mechanical allodynia in rats. The efficacy of the fucoidan-based cream at a high dose was comparable with the efficacy of diclofenac gel. The fucoidan-based cream could be considered a promising anti-inflammatory formulation.

## 1. Introduction

Marine-derived compounds demonstrate promising biological activities, and they are truly biodegradable and biocompatible, encouraging the development of novel formulations with specific pharmacological features of interest for the pharmaceutical industry. In particular, interest in the development of formulations with fucoidan has rapidly increased in the past few decades [1,2,3,4]. Fucoidan is a polysaccharide with glorious potential for pharmacological activities [5,6,7]. Among these, the anti-inflammatory properties of fucoidan have gained meaningful attention [8,9].

Inflammation is attracting the attention of the global scientific community since it is involved in various human diseases [10]. Due to the complexity of inflammation processes, there is an urgent need for the development of new and safe anti-inflammatory agents with multiple mechanisms of action. Anti-inflammatory mechanisms described for fucoidan include scavenging of free radicals [11,12], suppression of the production of nitric oxide, tumor necrosis factor-alpha (TNF-α), prostaglandin E2, interleukin-1 beta, and interleukin-6 [13,14], selective inhibition of cyclooxygenase-2 [12], and downregulation of the expression of mitogen-activated protein kinase p38, Akt, extracellular signal-regulated kinase (ENK), and c-Jun N-terminal kinase (JNK) [15,16].

The formulation of dosage forms plays a crucial role in the delivery, bioavailability, and stability of marine-derived compounds [17,18,19,20,21]. The topical application of drugs has several advantages compared to other routes of administration. Topically applied drugs avoid extensive first-pass metabolism, provide direct access and localization at the site of action, are usually well-tolerated, and can be an alternative for patients who cannot use other administration routes [22,23]. Today, formulations for topical application with local or systemic effects are mainly represented by semisolid preparations, such as ointments, creams, or gels [24]. Lipid-based formulations for dermal and transdermal drug delivery have steadily attracted attention in recent decades. Modern developments are focused on improving bioavailability, controlling drug release, and increasing the stability of the formulation [25].

This study aimed to develop and optimize a fucoidan-based cream formulation and investigate its anti-inflammatory potential after topical application in vivo.

## 2. Results and Discussion

### 2.1. Model Formulations with Fucoidan

Fucoidan does not form stable gels when mixed with water. Therefore, the addition of other polymers is required [26]. In the preliminary experiments, we tested the following excipients for the preparation of the cream base: natrosol, carbopol, Kolliphor P 407, Geleol, Gelucire 43/01, lanolin, and Cremophor A25. The aqueous solution of fucoidan forms a heterogeneous gel with cellulose derivatives natrosol and carbopol. Therefore, these compounds were excluded from future experiments. Olive oil was selected as the oil phase for the cream base. Olive oil easily penetrates the epidermis of the skin, ensuring good absorption of medicinal substances [27]. To stabilize the oil in water (o/w) emulsion, Kolliphor RH40 was added [28]. The concentrations of fucoidan and olive oil were derived from previous research [29]. Five model formulations (PF1–PF5) were prepared (Table 1).

### 2.2. Testing of Model Formulations

To assess the release of fucoidan from model formulations and the quality of formulations, the diffusion of fucoidan into an agar gel was studied. No diffusion of fucoidan from formulation PF4 with lanolin was observed for 18 h. The diffusion from formulations PF5 and PF3 was very insignificant (0.2 ± 0.1 mm and 1.0 ± 0.2 mm, respectively). On the other hand, diffusion from model formulations PF1 and PF2 started within the first hour; after 18 h, the zone was extended to 2.5 ± 0.1 mm.

**Figure 1:**
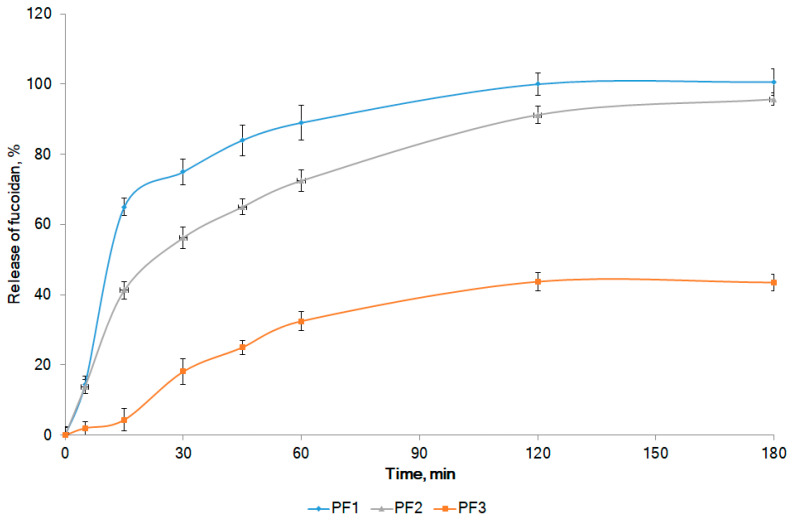
In vitro release profiles of fucoidan from model formulations. The compositions of the formulations are presented in Table 1.

The in vitro release profiles of fucoidan for model compositions are presented in Figure 1. Notably, the diffusion zones were correlated with the rate of release according to the following equation: *y* = 2.7563*x* + 0.1785 (*R*^2^ = 0.9527), where *x* is the diffusion in mm, and *y* is the rate of release in min^−1^.

Fucoidan was not released from formulations with lanolin (PF4) and Cremophor A25 (PF5) within 3 h (release rate 0.022 ± 0.006 and 0.104 ± 0.008 min^−1^, respectively; the release profiles are not shown in Figure 1). Although the above formulations had good washability (++), they showed relatively low spreadability (16.8% ± 0.9% and 20.1% ± 1.9 %, respectively), did not show colloidal stability, and did not pass the skin irritation test. Therefore, formulations PF4 and PF5 were not considered going forward. Formulation PF3 with Gelucire 43/01 did not provide a complete release of fucoidan. After 2 h of the experiment, about 43% of fucoidan was released (release rate 4.18 ± 0.06 min^−1^) with no future increase up to 3 h (Figure 1). This composition passed the skin irritation test; however, it did not exhibit colloidal stability, had poor washability (+), and showed low spreadability (14.9% ± 2.2%). The addition of geleol provided a sufficiently complete release of fucoidan (about 95% in 3 h) from composition PF2. This formulation showed colloidal stability and passed the skin irritation test, but it had poor washability (+). The spreadability (25.4% ± 0.5%) and fucoidan release rate (6.42 ± 0.16 min^−1^) were lower for PF2 when compared with the same parameters for PF1. The complete release of fucoidan was observed from formulation PF1 with Poloxamer 407 (Kolliphor^®^ P 407). After 1 h of the experiment, about 90% of the active substance passed into the dissolution medium. After 2 h, 100% of fucoidan was released (release rate 6.98 ± 0.17 min^−1^). Our results are consistent with [30], who reported a positive effect of Poloxamer 407 on stable gel formation with another polysaccharide typical for brown algae—alginate. The addition of Poloxamer 407 to hyaluronic acid improved the rheological properties of the gel formulation [31]. Composition PF1 was colloidally stable, was nonirritating to the skin, and showed excellent washability (+++) and good spreadability (31.6% ± 1.2%). This formulation was selected for future experiments.

### 2.3. The Effects of Permeation Enhancers and Emollients

The passive diffusion of drugs plays a main role in transdermal drug delivery. It can be enhanced by increasing the concentration of the drug in the formulation or by increasing skin permeability. Penetrants reversibly modify the structure of the stratum corneum and, as a consequence, weaken its barrier function [32]. Due to the hydrophilic properties of fucoidan, penetrants were selected from the group of hydrophilic nonionic surfactants, soluble or miscible with water. Dimethyl sulfoxide is one of the earliest and most widely studied penetration enhancers [32]. Transcutol P is a powerful solubilizing agent, and it is attractive as a penetration enhancer due to its lack of toxicity, biocompatibility with the skin, miscibility with polar and nonpolar solvents, and optimal solubilizing properties for several drugs [33]. Polysorbate 80 enhances drug diffusion, whereas, at concentrations above 5%, transdermal flux is decreased [34]. Different compositions with penetrants (dimethyl sulfoxide, Transcutol P, and polysorbate 80) were prepared using the optimized formulation PF1 (Table 2).

**Figure 2:**
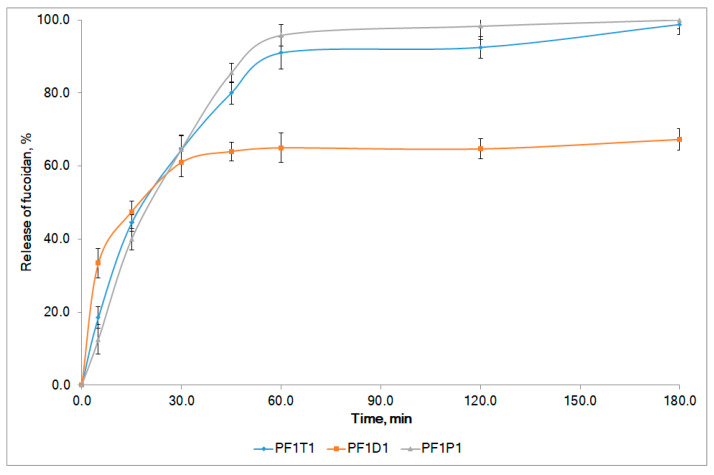
In vitro release profiles of fucoidan from model formulations with 1% penetrants. The compositions of formulations are presented in Table 3.

It was found that the addition of dimethyl sulfoxide as a penetrant slowed fucoidan diffusion into the agar gel and reduced the spreadability of the formulations when compared with PF1 (Table 3). Transcutol P and polysorbate 80 enhanced the diffusion of fucoidan from formulations into agar. However, the spreadability of formulations with Transcutol P was much better than that with polysorbate 80. An increase in the content of penetrants from 1% to 3% did not lead to a statistically significant increase in fucoidan diffusion and spreadability of formulations. Therefore, an in vitro dissolution test was performed for formulations with 1% penetrants (Figure 2).

About 65% of fucoidan was released from the composition with dimethyl sulfoxide (PF1D1) after the first hour, and no further release occurred in the next 2 h. This evidenced a low bioavailability of fucoidan from PF1D1. The use of polysorbate 80 (PF1P1) and Transcutol P (PF1T1) as penetrants provided a reasonable complete release of the active substance. After 1 h, about 90% of fucoidan was released from both formulations. The release rates were 6.75 ± 0.22 min^−1^ and 7.54 ± 0.19 min^−1^ for PF1T1 and PF1P1, respectively. The release profiles were similar (similarity factor f2 = 66.7, difference factor f1 = 5.7). Both formulations were involved in the next round of experiments.

Emollients are used to prevent trans-epidermal water loss, as well as provide skin hydration, lubrication, and penetration enhancement [35]. According to literature recommendations, PEG400 and glycerol were used as emollient enhancers [35,36]. Formulations PF1T1P5 and PF1P1T5 were prepared from PF1T1 and PF1P1, respectively, with the addition of 5% PEG400. Formulations PF1T1G5 and PF1P1G5 were prepared from PF1T1 and PF1P1, respectively, with the addition of 5% glycerol (Table 4).

All formulations were colloidally stable and did not irritate the skin. The addition of both emollients to the formulations with Transcutol P (PF1T1P5 and PF1T1G5) did not statistically significantly enhance the diffusion of fucoidan into the agar gel. On the other hand, the diffusion of fucoidan from the formulations with polysorbate 80 slightly decreased. Both emollients fortified the spreadability of formulations with Transcutol P (Table 4). Taking into account the largest diffusion zone and spreadability, formulation PF1T1P5 was most promising, representing a homogeneous cream of brown color with a specific odor.

### 2.4. Mechanisms of Fucoidan Release from the Optimized Formulation

**Figure 3:**
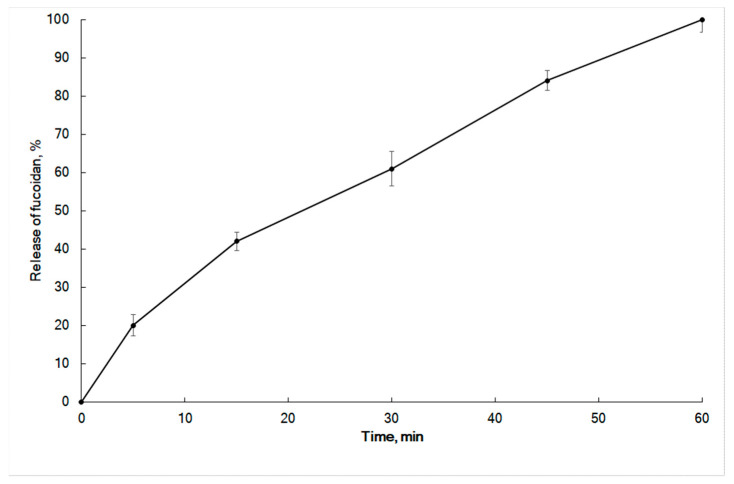
In vitro release profiles of fucoidan from the cream.

The results of in vitro release of fucoidan from the fucoidan-based cream are presented in Figure 3. Zero-order, first-order, Higuchi, Hixson–Crowell cube root law, and Korsmeyer–Peppas models [37,38] were tested to analyze the release mechanism of fucoidan. The in vitro release profile of fucoidan cream did not follow zero-order kinetics (Equation (1)), first-order kinetics (Equation (2)), or Hixson–Crowell cube root law (Equation (4)) (*R*^2^ < 0.98) (Table 5). On the other hand, the release profile of fucoidan fits well with Higuchi’s model (Equation (3)) (*R*^2^ = 0.9938). Thus, the drug release mechanism was assumed to be diffusion-controlled.

Numerous release processes can be described by a coupling of Fickian and non-Fickian mechanisms. Ritger and Peppas introduced the Korsmeyer–Peppas equation (Equation (5)) to characterize the controlled release behavior of a drug from matrices when the release mechanism is not well known or when more than one type of release phenomenon could be involved [39]. Peppas (1985) proposed an *n*-value to distinguish the release mechanisms. When *n* = 0.5, the drug is released from the matrix according to the quasi-Fickian diffusion mechanism, whereas *n* > 0.5 is evidence of anomalous, non-Fickian drug diffusion. In the case of *n* = 1.0, non-Fickian, zero-order release kinetics occurs [40]. The Korsmeyer–Peppas function described fucoidan release from the cream with a high correlation (*R*^2^ = 0.9981). The *n*-value of 0.65 (Table 5) indicates that the formulation exhibited anomalous transport (i.e., a non-Fickian diffusion mechanism). Taking together the results of Higuchi’s and Korsmeyer–Peppas models, we suggest that fucoidan release was due to both diffusions of the drug and anomalous transport provided by the optimized cream base.

### 2.5. Storage Stability

The data of stability studies on the fucoidan-based cream are summarized in Table 6. The concentration of fucoidan, its release, and the colloidal stability of the cream remained unchanged (*p* > 0.05, *n* = 6) after being stored in either ambient or cool conditions (5 ± 3 °C) for 365 days, thus demonstrating the high physical stability of the formulation.

### 2.6. Anti-Inflammatory Activity

**Figure 4:**
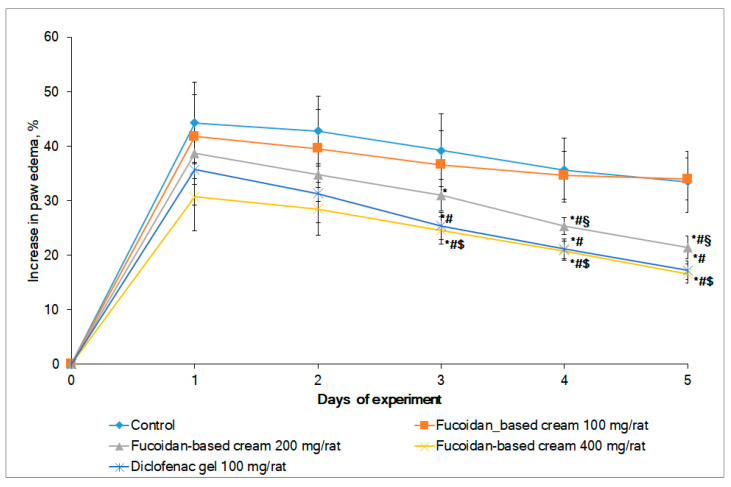
Dynamic of rat paw edema following topical application of fucoidan-based cream and diclofenac gel (*n* = 8 for all groups). ^#^ Significantly different compared with control (*p* < 0.05); * significantly different compared with the first day within the group (*p* < 0.05); ^§^ significantly different compared with fucoidan-based cream 100 mg/rat (*p* < 0.05); ^$^ significantly different compared with fucoidan-based cream 100 mg/rat and fucoidan-based cream 200 mg/rat (*p* < 0.05).

The anti-inflammatory activity of the developed fucoidan-based cream was assessed in two in vivo models: the model of carrageenan-induced rat paw edema and the mechanical allodynia test. Carrageenan-induced rat paw edema is a well-accepted model of inflammation recommended for the investigation of new anti-inflammatory drugs [41]. Female rats were involved in the study due to more pronounced signs of experimental pathology of inflammatory genesis when compared with male rats [42]. The injection of carrageenan to the hind paw led to a significant increase in paw volume. The fucoidan-based cream dose-dependently inhibited paw edema after topical application (Figure 4). A statistically significant inhibition of paw edema was observed starting from the third day of the experiment in both the diclofenac gel and the fucoidan cream groups (at 400 mg/rat) (analysis of variance (ANOVA) F test; *p* < 0.05). Interestingly, the inhibition of edema by the fucoidan-based cream (400 mg/rat) on the fifth day was over 50%. Notable, the efficacy of the fucoidan-based cream at the high dose was equal to the efficacy of diclofenac gel.

Our results are in line with previously published findings. The inhibition of carrageenan-induced edema was dose-dependent in rats after intraperitoneal injection of fucoidan from three species of *Cystoseira* brown alga [43]. A 51–58% inhibition was found at 50 mg/kg fucoidan from different species and was comparable to the efficacy of diclofenac. The onset of inhibition was rapid (1 h), and inhibition was observed for 5 h. Dose-dependent inhibition of carrageenan-induced rat edema by fucoidan from *Undaria pinnatifida* was reported after oral administration [44]. The maximum suppression of inflammation (68.19%) was observed 24 h after administration of 150 mg/kg of fucoidan. The authors associated the decrease in edema with the ability of fucoidan to inhibit IL-1β-induced COX-2 expression in chondrocytes.

**Figure 5:**
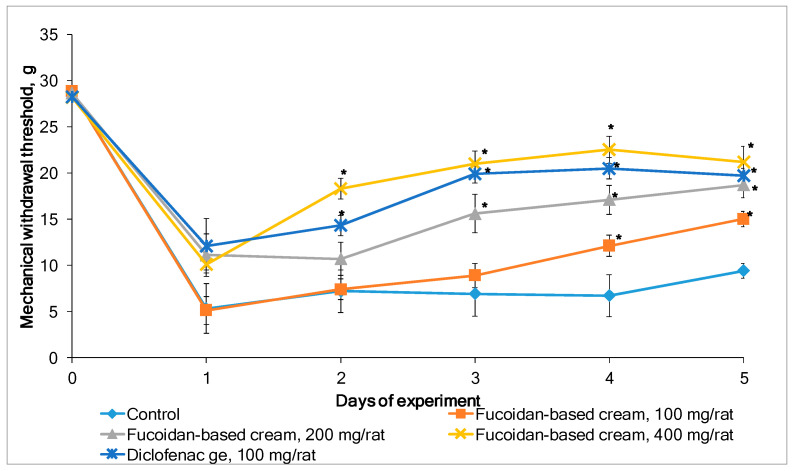
Effects of topical application of fucoidan-based cream and diclofenac gel on mechanical allodynia induced by carrageenan. Values are expressed as the mean ± SD (*n* = 8). * *p* < 0.05 compared with control (placebo).

Carrageenan-induced inflammation is accompanied by the development of pain syndrome. The mechanical withdrawal response of the inflamed rat paw in the von Frey hair model was used to investigate whether the fucoidan-based cream attenuates mechanical allodynia. Figure 5 shows the inflammatory pain responses (mechanical withdrawal threshold) in rats after topical application of a placebo (control group), fucoidan-based cream, or diclofenac gel. The repeated application of fucoidan-based cream for five consecutive days dose-dependently alleviated carrageenan-induced allodynia in rats. A statistically significant increase in paw withdrawal threshold was observed for the cream at a high dose (400 mg/rat) starting from the second day of the experiment. Although the effect of diclofenac gel was more prominent on the second day, starting from the third day, the efficacy of the fucoidan-based cream (400 mg/rat) was equal to the efficacy of diclofenac gel (*p* < 0.05). Noteworthily, no irritation or other adverse effects of the fucoidan-based cream were observed during the 5 days of treatment in both models of inflammation in rats.

Similar to the therapeutic effect of herbal extract with a prolonged onset, repeated application of fucoidan is required to produce antinociception. Lower doses of cream require additional time to onset the analgesia. Our results are consistent with those of other scientists, who reported the ameliorative effect of fucoidan on mechanical allodynia and thermal hyperalgesia [45] and mechanical and cold allodynia [46] after repeated intrathecal injection of fucoidan. Hu et al. (2014) associated the analgesic effect of fucoidan after intrathecal injection with inhibition of spinal astrocytic and microglial activation, proinflammatory mediator production, and mitogen-activated protein kinases (MAPK) activation. This hypothesis is supported by recent studies, which indicate suppression of the production of TNF-α, prostaglandins, and interleukins [13,14], as well as potent inhibition of kinases, including ENK, JNK, Akt [16], and MAPK p38 [12], by fucoidan. The rationality of topical application of formulations with fucoidan was confirmed in our recent study [47], in which fucoidan was found in the skin, plasma, and striated muscles. In contrast, the anti-inflammatory, antihyperalgesic, and antiallodynic effects of a phenolic-rich EtOH extract from *Posidonia oceanica* (POE) showed a rapid onset. POE dose-dependently counteracted carrageenan- and IL-1β-induced acute paw edema and inflammation pain in mice [48]. This suggests a more quick absorption of phenolics after peroral administration when compared with the transdermal absorption of polysaccharide fucoidan.

As far as we know, this is the first study in which the anti-inflammatory effects of a cream formulation with fucoidan from *Fucus vesiculosus* were investigated in a model of carrageenan-induced rat paw edema and mechanical allodynia in rats. Taking together our findings and the literature data, we believe that fucoidan can be regarded as a compound where the topical application is more favorable compared with injection due to ease to use, high patient compliance, and local activity. Due to its prolonged onset, we suggest that topical application of a fucoidan-based cream could be beneficial in the therapy of chronic inflammatory diseases. Future studies are required for confirmation.

## 3. Materials and Methods

### 3.1. Materials

Fucoidan with an average molecular weight of 735 kDa was provided by MMBI RAS (Murmansk, Russia). *F. vesiculosus* was collected from the littoral of the Barents Sea (Dalnie Zelentsy, Murmansk region, Russia) in August (fertility phase). Seaweed was washed with freshwater, frozen, and stored at −18 °C. Fucoidan was extracted as described previously [49]. Briefly, a frozen seaweed sample was ground and extracted with a mixture of methylene chloride/ethanol. After filtration, the residue was extracted in an ultrasound bath with a 5% aqueous solution of ethanol at 40 °C for 4 h at pH 3–4. The liquid fraction was isolated by centrifugation. After centrifugation, the crude fucoidan was dialyzed through a tangential membrane filter and freeze-dried. Fucoidan contained 79.5% of neutral carbohydrates, 27.0% of sulfate residues, and 0.7% of uronic acid. Carbohydrates were represented by fucose (73.5 mol.%), glucose (11.8 mol.%), galactose (3.7 mol.%), xylose (6.6 mol.%), mannose (0.2 mol.%), and arabinose (0.2 mol.%). The molar ratio of fucose, glucose, galactose, xylose, mannose, and arabinose was 1.0:0.16:0.05:0.09:0.03:0.03, respectively, as evidenced by high-performance liquid chromatography (HPLC) [12].

Poloxamer 407 (Kolliphor^®^ P 407), Polyoxyl 40 hydrogenated castor oil (Kolliphor^®^ RH40), and polyethylene glycol 400 (Kollisolv^®^ PEG 400) were provided by BASF (Ludwigshafen, Germany). Extra-virgin olive oil was purchased at a local market. Diethylene glycol monoethyl ether (Transcutol^®^ P), mono and diglycerides NF (Geleol^®^), and mixtures of mono-, di-, and triglycerides (Gelucire^®^ 43/01) were gifts from Gattefossé (Saint-Priest, France). Polyoxyethylene 20 Sorbitane mono-oleate (Tween^®^ 80) and glycerol were from PanReac Applichem (Barcelona, Spain). λ-Carrageenan (Type IV) was obtained from Sigma-Aldrich (St. Louis, MO, USA). Other materials used in the study (fucose, l-cysteine hydrochloride, concentrated sulfuric acid, etc.) were of analytical grade.

### 3.2. Preparation of Cream Formulations

Fucoidan-containing cream formulations were prepared using olive oil and additional modifying excipients of various natures (Table 1). Aqueous and oil phases were prepared separately. Fucoidan and Kolliphor^®^ P 407 were dissolved in water. Lipophilic components (Kolliphor^®^ RH40, Geleol, Gelucire 43/01, lanolin, and Cremophor^®^ A25) were dissolved in olive oil. Both phases were mixed at room temperature with stirring. The excipients were selected based on the literature data [50,51,52] and the authors’ experience.

### 3.3. Diffusion in Agar

Diffusion in agar was used as an alternative method for studying drug release according to [53]. Briefly, the agar gel was poured into Petri dishes, and a stainless-steel cylinder (diameter 9 mm) was preliminarily placed in each dish. The thickness of the agar gel layer was 8 mm. After the agar solidified, the cylinders were carefully removed. Weighed portions of the cream samples (about 170 mg) were placed in the wells. The dishes were placed in a desiccator with a temperature of 32 °C for 18 h. Diffusion was assessed by the change in the staining zone.

### 3.4. Fucoidan Release In Vitro

The fucoidan release was studied using the USP 23 apparatus 5, featuring a paddle over a disc, with 500 mL of dissolution medium (water) at 32 ± 1 °C and a stirring speed of 100 rpm. Samples (2 mL) were removed at predetermined time intervals (5, 15, 30, 45, 60, 120, and 180 min), filtered, and assayed by a spectrophotometric method according to the reaction of fucose with l-cysteine [54] with slight modification [55]. Briefly, 4 mL of sulfuric acid solution (concentrated sulfuric acid–water, 5:2 *v*/*v*) was added to 0.5 mL of the test solution and stirred. Then, 1 mL of concentrated sulfuric acid was added and stirred, before heating in a water bath for 10 min. Next, 0.1 mL of a 3% solution of l-cysteine hydrochloride was added and stirred, kept before being maintained for 2 h. The optical densities of the solution were measured on a UV-1700 mini spectrophotometer (Shimadzu, Japan) at 396 nm. The reference solution was a mixture consisting of 0.5 mL of water, 4 mL of the sulfuric acid solution, and 1 mL of concentrated sulfuric acid, treated similarly to the test solution. In parallel, the optical density of a solution of a standard sample of fucose, treated similarly to the test solution, was measured. An equal volume of fresh medium was immediately added to maintain the dissolution volume. Absorbance was converted to drug concentration using the linear equation of a calibration curve, and then the cumulative percentage of fucoidan released was calculated taking into consideration the dilution factor. All measurements were performed in triplicate (*n* = 3). The calibration curve for fucose was linear over a concentration range of 0.0077–0.077 mg/mL (*R*^2^ = 0.998).

### 3.5. Spreadability, Washability, Colloidal Stability, and Skin Irritation Tests

The spreadability was analyzed according to [56] with minor modification. Briefly, about 1.0 g of composition (Table 1) was placed on a glass plate (10 × 10 cm). Another glass plate was placed on top of the sample, after which it was loaded with weights of increasing mass (50, 100, 200, and 1500 g) at intervals of 1 min. The diameters of the spots were recorded at each interval. The spreadability was calculated as a function of the spot diameter after loading of weights, expressed as a percentage. Data were the average of three determinations at 25 °C.

In the washability test, compositions were applied on the skin, and then the easy method of washing with water was applied [57]. Results were denoted as “+++” (good, does not stick to unprotected human skin), “++” (sticks slightly to unprotected human skin), or “+” (sticks to unprotected human skin).

For colloidal stability, 0.2 g of the composition was placed in a test tube in a water bath for 20 min at 42–45 °C. The test was performed using a laboratory centrifuge for 5 min with a speed of 5000 rpm. The sample was considered stable if, after centrifugation in test tubes, no more than one drop of the aqueous phase was observed [58].

For the skin irritation test, compositions prepared were applied to the skin of human beings and observed for the effect [59].

### 3.6. Analysis of Release Profiles

The data of fucoidan release from the formulations were evaluated using the DDSolver Excel add-in [60]. The similarity factor (f2) was defined as the logarithmic reciprocal square root transformation of one plus the mean squared (the average sum of squares) differences of the drug percentage dissolved between two test formulations [61].

The zero-order model illustrates systems where the rate of drug release is independent of the initial concentration of the drug [62].

Zero-order equation:

(1) $$Q_{t}=k_{0}\cdot t,$$

where $Q_{t}$ is the percentage of drug released at time *t*, and *k*_0_ is the release rate constant.

First-order equation [62]:

(2) logQ=logQ0−k1·t2.303,

where *Q*_0_ is the initial concentration of the drug, *k*_1_ is the first-order rate constant, and *t* is the release time.

Higuchi’s equation is the most widely used model to describe drug release from pharmaceutical matrices [62].

(3) $$Q_{t}=k_{H}\sqrt{t},$$

where *Q_t_* is the amount of drug released at time *t*, and *k_H_* is the Higuchi release rate.

Hixson–Crowell cube root law [62]:

(4) $$Q_{0}^{1/3}-Q_{t}^{1/3}=K_{HC}\cdot t,$$

where *Q_t_* is the amount of drug released in time *t*, *Q*_0_ is the initial amount of drug in formulation, and *K_HC_* is the rate constant for the Hixson–Crowell rate equation.

The Korsmeyer–Peppas semiempirical model was also applied [39].

(5) $$\frac{Q_{t}}{Q_{\infty }}=K\cdot t^{n},$$

where *Q_t_/Q_∞_* is the fractional drug release from the matrices into the dissolution medium, *K* is a constant corresponding to the structural and geometric characteristics of the device, and *n* is the release exponent which is indicative of the mechanism of the drug release [40].

### 3.7. Storage Stability Test

The optimized formulation was packed in glass vials with caps and stored in ambient conditions and at 5 ± 3 °C for 365 days. The samples were tested at 0, 90, 180, 270, and 365 days. The formulation was analyzed for colloid stability, drug content, and drug release.

### 3.8. Anti-Inflammatory Activity

The activity was tested on Wistar albino rats (Rapplovo animal house, St. Petersburg, Russia). The animals were housed in plexiglass cages with free access to food and water and maintained at a constant temperature of 19–25 °C and relative humidity with a 12 h light/dark cycle. After a 2 week adaptation period, 40 female rats (about 250–330 g) were assigned randomly to four groups (*n* = 8). For the induction of edema, all animals were injected subcutaneously into the plantar region of the right hind paw with 0.1 mL of 3% carrageenan. The first (control group) was not treated. The second group (positive control) was treated with 100 mg/rat of diclofenac gel 1% (Akrikhin JSC, Moscow, Russia). The other three groups received 100, 200, and 400 mg/rat of fucoidan-based cream. The daily dose of tested drugs was divided into two applications (50% morning and 50% evening, at least 7 h apart). The first application was made 30 min after injection of carrageenan. Experiments were performed for 5 days. The rat paw volume was measured using the oncometric method [63] daily, 1 h after the morning drug application. The data are presented as an increase in paw edema [64].

Mechanical allodynia was assessed using calibrated von Frey hairs (North Coast Medical Inc., Morgan Hill, CA, USA) [45]. The stiffness of the von Frey hairs was 0.692, 1.202, 2.041, 3.630, 5.495, 8.511, 15.136, and 28.840 g. A positive paw withdrawal response was recorded if the animal briskly lifted its hind paw. The mechanical withdrawal threshold was determined using the up-down method [65]. The placebo cream was prepared using all components except fucoidan.

### 3.9. Statistical Analysis

Experimental results are expressed as means ± standard deviation (SD). Statistical significance was assessed as *p* < 0.05. Data were analyzed using Statistica version 6.0.

## 4. Conclusions

The topical route of drug application has great potential as an effective and safe method of drug delivery. In this study, the development of cream formulations with fucoidan from brown algae *Fucus vesiculosus* is described. The cream base consisting of olive oil and Kolliphor RH40 was optimized, followed by in vitro agar diffusion and drug release studies. The fucoidan-based cream with 13% Kolliphor P 407, 1% Transcutol P, and 5% PEG400 showed good spreadability, washability, and colloidal stability, and it did not irritate the skin. The kinetics of fucoidan release from optimized cream exhibited the best fit to the Korsmeyer–Peppas and Higuchi models with *R*^2^ > 0.99. Our data suggest that fucoidan release was controlled by drug diffusion and anomalous transport provided by the optimized cream base. The formulation was stable and provided high fucoidan release after storage for 1 year. In vivo experimental results suggested that topical application of the fucoidan-based cream dose-dependently inhibited carrageenan-induced edema and ameliorated mechanical allodynia in rats. The efficacy of the fucoidan-based cream at a high dose was comparable with the efficacy of diclofenac gel. The fucoidan-based cream could be considered a promising anti-inflammatory formulation.

## Figures and Tables

**Table 1:** Model formulations (*w*/*w*, %) with fucoidan.

Compounds	Formulation Code				
	PF1	PF2	PF3	PF4	PF5
Fucoidan	15	15	15	15	15
Olive oil	10	10	10	10	10
Kolliphor^®^ RH40	8	8	8	8	8
Kolliphor^®^ P 407	13	0	0	0	0
Geleol	0	5	0	0	0
Gelucire 43/01	0	0	15	0	0
Lanolin	0	0	0	30	0
Cremophor^®^ A25	0	0	0	0	3
Water up to	100	100	100	100	100

**Table 2:** Formulations of fucoidan based on PF1 with permeation enhancers (*w*/*w*, %).

Penetrant	Formulation Code					
	PF1T1	PF1T3	PF1D1	PF1D3	PF1P1	PF1P3
Dimethyl sulfoxide	0	0	1	3	0	0
Transcutol P	1	3	0	0	0	0
Polysorbate 80	0	0	0	0	1	3

**Table 3:** Diffusion zones and spreadability of formulations with permeation enhancers.

Parameter		Model Formulations				
	PF1T1	PF1T3	PF1D1	PF1D3	PF1P1	PF1P3
Diffusion zone, mm	3.2 ± 0.3	3.4 ± 0.2	2.0 ± 0.2	2.0 ± 0.1	3.0 ± 0.4	3.2 ± 0.3
Spreadability, %	47.5 ± 3.0	51.7 ± 2.8	26.2 ± 4.2	24.8 ± 4.1	36.1 ± 1.4	36.5 ± 1.9

**Table 4:** Diffusion zones and spreadability of formulations with emollients.

Parameter	Model Formulations			
	With 5% PEG400		With 5% Glycerol	
	PF1T1P5	PF1P1P5	PF1T1G5	PF1P1G5
Diffusion zone, mm	3.5 ± 0.2	2.5 ± 0.3	3.1 ± 0.2	2.5 ± 0.3
Spreadability, %	55.0 ± 1.4	33.9 ± 1.2	50.7 ± 1.8	32.7 ± 3.2

**Table 5:** Release parameters of fucoidan from cream.

Zero-Order		First-Order		Higuchi		Hixson–Crowell Cube Root Law		Korsmeyer–Peppas		
k_o_	*R* ^2^	k_1_	*R* ^2^	k_H_	*R* ^2^	K_HC_	*R* ^2^	K_kp_	*n*	*R* ^2^
1.82	0.8262	0.038	0.9385	12.13	0.9938	0.01	0.9415	7.06	0.65	0.9981

**Table 6:** Stability study of fucoidan-based cream in cool and ambient conditions.

Storage Conditions	Parameter	Days				
		0	90	180	270	365
1	Fucoidan content, %	100.0 ± 2.7	99.7 ± 3.0	99.0 ± 2.4	98.4 ± 2.9	98.0 ± 3.6
	Fucoidan release at 1 h, %	100.0 ± 2.8	100.0 ± 2.9	99.6 ± 3.6	98.9 ± 3.2	98.5 ± 3.9
	Colloidal stability	Stable	Stable	Stable	Stable	Stable
2	Fucoidan content, %	100.0 ± 2.2	99.9 ± 3.6	100.0 ± 3.1	99.5 ± 2.7	99.2 ± 2.5
	Fucoidan release at 1 h, %	100.1 ± 3.0	101.2 ± 2.9	99.6 ± 3.9	99.3 ± 3.0	98.9 ± 2.7
	Colloidal stability	Stable	Stable	Stable	Stable	Stable

Storage condition: 1, ambient conditions; 2, cool conditions (5 ± 3 °C).

## Data Availability

Data is contained within the article.
